# Visible and rapid detection of feline chaphamaparvovirus using multienzyme isothermal rapid amplification and lateral flow dipstick assay

**DOI:** 10.3389/fcimb.2025.1490948

**Published:** 2025-01-22

**Authors:** Jun Ji, Xinhao Mu, Shunshun Pan, Xin Xu, Shiyuan Zhang, Honghui Huang, Ying Li, Yingzuo Bi, Lunguang Yao

**Affiliations:** ^1^ Henan Provincial Engineering and Technology Center of Animal Disease Diagnosis and Integrated Control, Henan Key Laboratory of Insect Biology in Funiu Mountain, Nanyang Normal University, Nanyang, China; ^2^ College of Animal Science and Veterinary Medicine, South China Agricultural University, Guangzhou, China

**Keywords:** FeChPV, MIRA-LFD, detection, sensitivity, simplicity

## Abstract

Feline chaphamaparvovirus (FeChPV) is a novel parvovirus previously reported in Canadian cats and Chinese dogs with diarrhea in 2019 and 2020, respectively. Herein, we aimed to establish a simple detection method for FeChPV in field clinics. The primers and probes for the multienzyme isothermal rapid amplification and lateral flow dipstick (MIRA-LFD) assay were designed to target the conserved regions of the FeChPV genome and determine the optimal reaction temperature and time. Without relying on precision instruments, FeChPV detection using the MIRA-LFD assay was completed within 20 min at 37°C, without any cross-reaction with other reference viruses. The newly established MIRA-LFD assay had a detection limit of 32.3 copies/μL, which was 10-fold lower than that of the nested polymerase chain reaction (PCR) assay. Furthermore, the MIRA-LFD assay detected 29 FeChPV-positive samples among 417 cats with diarrhea, providing a slightly higher positivity rate than the nested PCR assay. These results indicate that the newly developed MIRA-LFD assay for FeChPV detection is an efficient, economical, reliable, and simple method that can help in the early prevention and control of FeChPV infection.

## 1 Introduction

Viruses belonging to the *Parvovirinae* family are unenveloped icosahedral virions harboring linear single-stranded DNA genomes with a size of 3.9–6.3 kb (Cotmore and Tattersall, 2014). According to the recent classification standards of the International Committee on Taxonomy of Viruses, the family *Parvoviridae* is classified into three subfamilies: *Densovirinae*, *Hamaparvovirinae*, and *Parvoviridae*. The *Hamaparvovirinae* subfamily includes the genera *Brevihamaparvovirus*, *Chaphamaparvovirus*, *Hepanhamaparvovirus*, *Ichthamaparvovirus*, and *Penstylhamaparvovirus* (Cotmore et al., 2014; Pénzes et al., 2020).

Chaphamaparvovirus (ChPV), which belongs to the subfamily *Hamaparvovirinae*, has been detected in various animals, including dogs, cats, rats, bats, owls, peacocks, mice, tilapia, red-crowned cranes, pigs, ducks, and chickens (Baker et al., 2013; Mohamed et al., 2013; Yang et al., 2016; Chong et al., 2019; Du et al., 2019; Lima et al., 2019; Wang et al., 2019; Ge et al., 2020; Liu et al., 2020; Vibin et al., 2020; Hargitai et al., 2021; Di Profio et al., 2022). Originally, feline chaphamaparvovirus (FeChPV) was detected in fecal samples obtained from an outbreak of feline acute gastroenteritis in Canada in 2019. Since then, it has been suspected to cause diarrhea and vomiting (Li et al., 2020). The FeChPV genome is approximately 4.3 knt long and contains three open reading frames: nonstructural protein 1 (NS1), virion protein 1 (VP1), and nuclear phosphorylated protein (Li et al., 2020). As a novel parvovirus, FeChPV has been detected in cats in Canada, Italy, and Turkey as well as in both cats and dogs in China (Abayli and Can-Sahna, 2022; Di Profio et al., 2022; Cui et al., 2023a). Unlike most ChPVs in other species, FeChPV has been frequently detected not only in the fecal samples of dogs and cats with diarrhea; but also in Chinese cats with respiratory diseases (Di Profio et al., 2022; Hao et al., 2022). However, its pathogenicity and molecular characterization remain poorly explored, necessitating continuous epidemic investigation for a deeper understanding.

The potential impact of FeChPV on fields remains unclear. As FeChPV is a potential enterovirus, an effective detection method is needed for early prevention and control. Currently, there is no standard assay for detecting FeChPV (Liu et al., 2022). The virus has not been successfully isolated *in vitro*, and no serological tests to detect antigens and antibodies have been established for FeChPV (Cui et al., 2023a). Therefore, molecular biological methods are currently the preferred choice for the detection of FeChPV DNA. As a widely used method for FeChPV detection, traditional polymerase chain reaction (PCR) requires precision instruments that are more suitable for laboratory testing alone (Li et al., 2020). Nested PCR (nt-PCR) has been successfully used for FeChPV detection with improved sensitivity; however, it requires accurate temperature control of the instrument (Di Profio et al., 2022). The steps involved in nucleic acid electrophoresis technology are technically demanding, time-consuming, and laborious and constrain the large-scale application of nt-PCR in field clinics. Furthermore, SYBR Green I and TaqMan-based quantitative PCR (qPCR) tests have been developed for FeChPV detection with certain sensitivity and specificity; however, they require advanced real-time measurement instruments and professional training, which are also only suitable for laboratory testing and not for large-scale rapid detection in field clinics (Di Profio et al., 2022; Liu et al., 2022). Therefore, it is urgent to establish an effective, rapid, convenient, and economical testing method.

Multienzyme isothermal rapid amplification (MIRA) achieves rapid and reliable amplification of target genes owing to the synergistic activity of helicase, recombinase, single-stranded binding protein, and DNA polymerase (Heng et al., 2022). The amplicons can be visually assessed using the lateral flow dipstick (LFD) assay to obtain the results within 5 min based on the control and test bands (Park and Chang, 2022). The MIRA-LFD assay has been developed for the detection of multiple viruses, including human hepatitis A virus, chicken chaphamaparvovirus, and bovine coronavirus (Sun et al., 2021; Ji et al., 2022; Cui et al., 2023b; Sun et al., 2023). Consequently, we aimed to establish the MIRA-LFD assay for FeChPV detection as an efficient and economical high-volume field detection method. Considering the genomic differences among different ChPVs, the MIRA-LFD assay established in this study is specific only to FeChPV. Thus, the successful establishment of this detection method will also provide a reference for the rapid detection of other ChPVs.

## 2 Materials and methods

### 2.1 Clinical samples and nucleic acid extraction

The rectal swabs used in this study were collected from 632 cats (417 cats with diarrhea and 115 healthy cats) and 474 dogs (342 dogs with diarrhea and 132 healthy dogs) at pet hospitals in Guangdong, Henan, Anhui, Zhejiang, and Inner Mongolia provinces from 2022 to 2024. Each sample was suspended in phosphate-buffered saline. Viral DNA and RNA were subsequently extracted from the samples using a viral DNA/RNA kit (TransGenBiotech, Beijing, China) following the manufacturer’s instructions, and the obtained DNA/RNA was stored at −80°C until use.

### 2.2 Primer and probe design for MIRA

Using an online software (https://www.ezassay.com/primer), four specific primer sets for MIRA targeting the conserved sequences (the overlapping region of NS1 and VP1) of strain IDEXX-1 (FeChPV prototype strain, accession no.: MN396757) were designed according to technical principles. Then, they were synthesized by Sangon Biotech Company (Zhengzhou, China). All reverse primers were labeled with biotin, an antigenic marker at the 5′-end that binds to a biotin ligand on the LFD. The specific amplicons carry both FAM and biotin haptens for binding to the T-line, where the biotin–antibody complexes have been immobilized.

To combine the LFD and MIRA assays, two probes (Probe1 for FeChPV-1/2 primer sets, and Probe2 for FeChPV-3/4 primer sets) were designed to match the primer sets of the upstream and downstream primer-targeting regions. The primer pairs and FAM-labeled probes for MIRA were optimized by analyzing the amplified products using 2% agarose gel electrophoresis and LFD (Amp-Future, Weifang, China). The primer sets and probes used in this study are listed in **Supplementary Table 1**.

### 2.3 Establishment of MIRA-LFD reaction systems

The MIRA reaction system (50 μL) included buffer A (29.4 μL), DNA extract of CHN201109 [accession no.: OQ694033 (Cui et al., 2023a)] or deionized water (for non-target control) (1.0 μL), buffer B (2.5 μL), and each set of primers (0.25 μL). Each reaction unit was supplied with a MIRA kit (Ampu Future Biotechnology, Ltd., Changzhou, China). For 2% agarose gel electrophoresis and imaging, the MIRA reaction products were mixed with the DNA extraction reagent (1:6) supplemented with DNA loading buffer. To perform MIRA testing with LFD (MIRA-LFD), 0.6 μL of probe (10 mM) was mixed with the MIRA reaction mixture, as described above. The MIRA products were considered negative or positive based on the LFD assay.

### 2.4 Optimal reaction time and temperature

To obtain a suitable MIRA assay, reaction conditions were optimized in a metal bath with gradient temperatures of 36°C, 37°C, 38°C, 39°C, and 40°C and gradient times of 5, 10, 15, and 20 min according to suggested reaction temperature and time of MIRA kit instructions. The reaction products were separated via agarose gel (2%) electrophoresis and analyzed using gel imaging systems.

### 2.5 Sensitivity analysis of MIRA-LFD

Standard plasmids (p-CHN201109) containing the genome sequence of CHN201109 [accession no.: OQ694033 (Cui et al., 2023a)] were used to determine the sensitivity of MIRA-LFD for FeChPV detection. A 10-fold gradient of p-CHN201109 (initial copy number of 3.23 × 10^6^) was sequentially diluted to determine the minimum detection limit for both MIRA-LFD and hemi-nt-PCR assays reported previously. This was achieved using forward primers (FechaF1: 5′-GGTGCGACGACGGAAGATAT-3′, FechaF2: 5′-GCTGCAGTTCAGGTAGCTCA-3′) and universal reverse primer (FechaR1: 5′-CAACACCACCATCTCCTGCT-3′) to amplify a 310-bp region (Li et al., 2020). The cycling conditions for nt-PCR were as follows: predenaturation at 95°C for 3 min, 35 cycles of denaturation at 94°C for 30 s, annealing at 54°C for 30 s, extension at 72°C for 30 s, and final extension at 72°C for 10 min. All detection limit tests for MIRA-LFD and nt-PCR were performed in 20 replicates. A probit analysis for determining the detection limit of MIRA-LFD and nt-PCR was performed at a 95% probability level. The detection limits of MIRA-LFD and nt-PCR were compared using SPSS software. *p*-values of <0.05 were considered to indicate statistical significance.

### 2.6 Specificity analysis of MIRA-LFD

The specificity of the MIRA-LFD detection method was analyzed by comparing the test strip results with LFD bands and via 2% agarose gel electrophoresis. This comparison was performed using positive nucleic acid samples of FeChPV, feline parvovirus (FPV), feline astroviruses (FeAstV), feline bocavirus (FBoV), and canine chaphamaparvovirus (CachaV), which were preserved in our laboratory.

### 2.7 Clinical sample detection

To further evaluate the effectiveness and accuracy of MIRA for FeChPV detection, the nt-PCR and MIRA-LFD assays were used to detect FeChPV in 1006 clinical samples, and the positive detection rates of the two methods were compared. The diagnostic results of the two detection methods were analyzed using SPSS software to evaluate the reliability of MIRA-LFD in clinical testing. The samples tested positive for FeChPV were sent to Suzhou Hongxun Company for sequencing to verify the presence of FeChPV and determine the existence of false-positive results.

## 3 Results

### 3.1 Primer and probe screening

**Figure 1:**
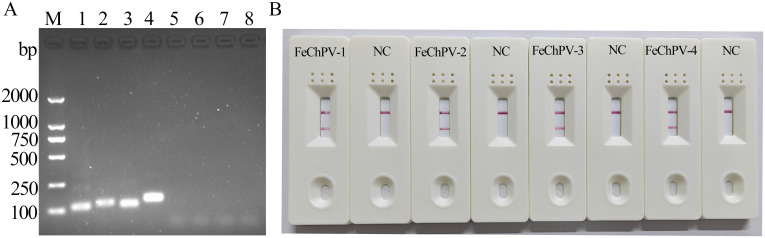
Screening primer pairs for FeChPV-MIRA. **(A)** Lanes 1, 2, 3, and 4 represent the results for four pairs of specific primers (amplified bands of 112, 128, 113, and 142 bp), and lanes 5, 6, 7, and 8 represent the corresponding negative controls of the four primer pairs; M: DNA molecular standard weight (DL2000). **(B)** MIRA-LFD results; NC: negative control.

The screening of the four designed primer pairs using gel electrophoresis and LFD showed that all four primer pairs can detect FeChPV DNA, and the products of MIRA-FeChPV-4 primer sets exhibited the brightest amplified bands (**Figure 1**). Therefore, MIRA-FeChPV-4 was explicitly used as a preferred primer pair for subsequent assays.

### 3.2 Optimal reaction time and temperature

**Figure 2:**
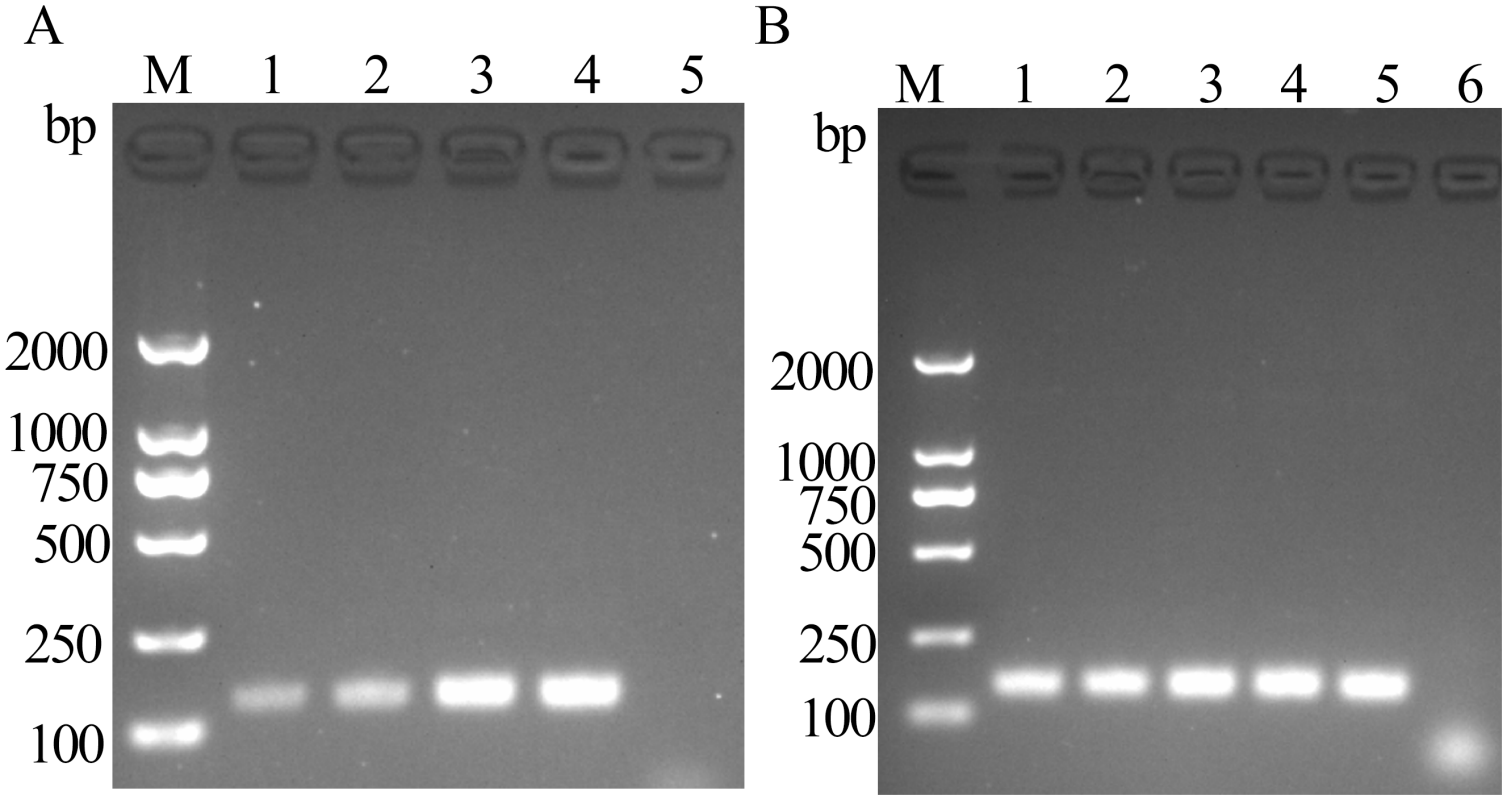
**(A)** Optimal time for MIRA reaction (amplified band of 142 bp); 1: 5 min, 2: 10 min, 3: 15 min, 4: 20 min, and 5: negative control; M: DNA molecular standard (DL2000). **(B)** Optimal temperature for MIRA reaction (amplified band of 142 bp); 1: 36°C, 2: 37°C, 3: 38°C, 4: 39°C, 5: 40°C, 6: negative control; M: DNA molecular standard DL2000.

The product bands appeared after 5 min of reaction, with the band intensity peaking at 15 min. According to gel electrophoresis results, the preferred optimal reaction time for FeChPV-MIRA was 15 min (**Figure 2A**). The target amplified bands were observed at 36°C–40°C, but the target band at <39°C was the brightest, making 39°C the optimal temperature (**Figure 2B**).

### 3.3 Sensitivity of the MIRA-LFD assay

**Figure 3:**
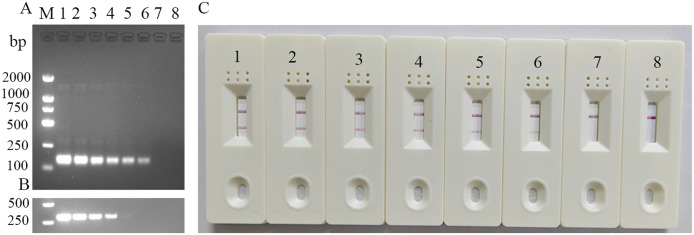
**(A)** Sensitivity results of MIRA-LFD for detecting FeChPV (amplified band of 142 bp); 1–7: FeChPV plasmid template: 3.23 × 10^6^ to 3.23 copies, 10-fold sequential gradient dilution, 8: negative control, M: DNA molecular standard DL2000. **(B)** Results of the nt-PCR electrophoresis of FeChPV (amplified band of 310 bp). 1–7: FeChPV positive plasmid template: 3.23×10^6^ to 3.23 copies, 10-fold sequential gradient dilution, 8: negative control, M: DNA molecular standard DL2000. **(C)** MIRA sensitivity lateral flow test strip results; 1–7: Gradient diluted FeChPV plasmid template, 8: negative control.

Serially diluted standard templates (p-CHN201109 plasmid) were detected using the established FeChPV-MIRA and nt-PCR assays (**Figure 3**). The LFD results were consistent with those of MIRA electrophoresis, with a detection limit of approximately 3.23 × 10 copies (probit analysis, *p* < 0.05). The detection limit of the nt-PCR assay was approximately 3.23 × 10^2^ copies (probit analysis, *p* < 0.05). Therefore, the sensitivity of the MIRA assay was approximately 10 times higher than that of the nt-PCR assay for FeChPV detection (*p* < 0.05).

### 3.4 Specificity of the MIRA-LFD assay

**Figure 4:**
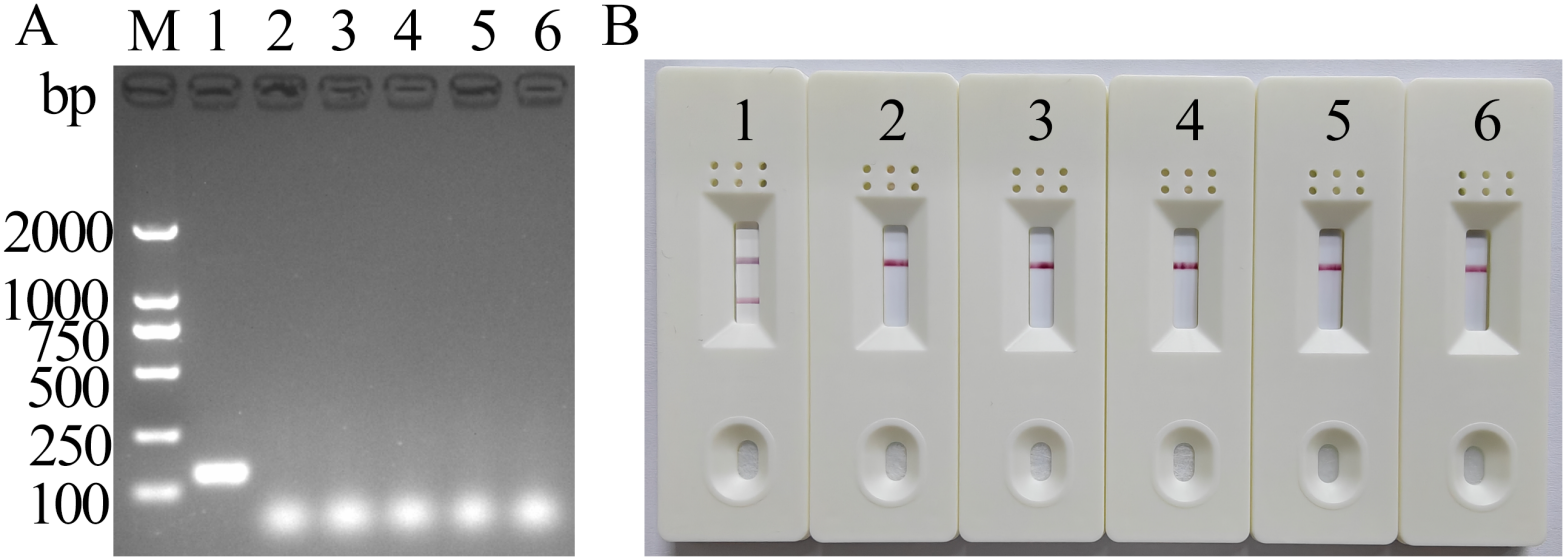
MIRA electrophoresis and LFD-specific results of FeChPV (amplified band of 142 bp). **(A)** Gel imaging results after electrophoresis. **(B)** Visualization results using LFD; 1: FeChPV, 2–5: FPV, FeAstV, FBoV, and CachaV; 6: negative control; M: DNA molecular standard DL2000.

The results of MIRA electrophoresis were consistent with those of LFD (**Figure 4**), suggesting that the newly established MIRA-LFD assay detected only FeChPV and displayed no cross-reactivity with other reference viruses, indicating its good specificity.

### 3.5 Detection of clinical samples

As shown in **Supplementary Table 2**, 36 of the 1006 collected rectal swabs were positive for FeChPV (3.57%) according to nt-PCR assay, and 38 were positive for FeChPV (3.77%) according to MIRA-LFD assay. Moreover, statistical analysis indicated high agreement between the two methods. The results also showed that the MIRA-LFD assay had higher sensitivity than nt-PCR.

## 4 Discussion

Previously, FPV was primarily regarded as the main causative agent of diarrhea in cats (Chang et al., 2020), whereas other viruses associated with feline diarrhea were overlooked (Li et al., 2018). However, enteric viruses such as FCV, feline astrovirus, and FBoV have drawn increased public attention in recent years (Di Martino et al., 2020; Wang et al., 2021). Between 2019 and 2020, FeChPV (36.8%, 14/38) was detected in Italian cats with upper respiratory diseases and diarrhea, with a higher detection rate and higher positive rate than FPV (23.7%, 9/38), feline coronavirus (5.3%, 2/38), and norovirus (5.3%, 2/38). Epidemic reports of FeChPV in southern, eastern, and central China showed that unlike other DNA viruses, FeChPV may have host and genetic diversity (Di Martino et al., 2019; Hao et al., 2022; Cui et al., 2023b). Notably, FeChPV was also found in the feces of healthy Turkish cats in 2022 (Abayli and Can-Sahna, 2022).

Since its first report in 2019, FeChPV has been suspected to be associated with diarrhea in cats (Li et al., 2020; Di Profio et al., 2022). However, owing to inadequate vaccines, prevention and control efforts mainly depend on early diagnosis. As reported in the literature, nt-PCR assay has been successfully used to detect FeChPV; however, it requires precise temperature control of instruments, and nucleic acid electrophoresis is technically demanding, time-consuming, and laborious. Therefore, nt-PCR assay is unsuitable for application in field clinics (Cui et al., 2023a).

The MIRA-LFD assay was developed for detecting human hepatitis A virus, chicken chaphamaparvovirus, and bovine coronavirus, and the method was reported to be simple and rapid (Sun et al., 2021; Ji et al., 2022; Cui et al., 2023b; Sun et al., 2023). The MIRA-LFD assay newly developed in this study exhibited an optimal temperature of 39°C. Intriguingly, the reaction temperature of 36°C–40°C indicates that the reaction can be performed at body temperature in various areas, including the fist and armpits, further reducing the reliance on the device. In this study, we attempted to perform clinical sample testing in the fist, with 28 of the 29 positive diarrheal samples in cats displaying a detection band, indicating high agreement. The MIRA-LFD assay only detected FeChPV, avoiding cross-reaction with other common feline and canine enteroviruses. For sensitivity evaluation, the MIRA-LFD assay was approximately 10 times more sensitive than the nt-PCR assay. In clinical testing, statistical analysis showed high concordance between the results of MIRA-LFD and nt-PCR. In total, 968 samples were detected as FeChPV-negative based on the two assays. Furthermore, 36 FeChPV-positive samples were detected by the two assays, which contained positive diarrheal samples and healthy samples from cats and dogs. Two positive diarrheal samples from cats were only detected by MIRA-LFD. All positive samples were confirmed through amplicon sequencing using the detection results of the MIRA-LFD assay. Additionally, the test results further proved that the sensitivity of the MIRA-LFD assay was higher than that of nt-PCR. The nt-PCR assay has been previously used for FeChPV detection, and its results were analyzed via gel electrophoresis and imaging. Therefore, in this study, we only compared the MIRA-LFD and nt-PCR assays for clinical testing. Based on previous studies, the minimum detection limit of the MIRA-LFD assay is similar to those of TaqMan real-time qPCR and SYBR Green I-based qPCR assays for FeChPV detection (Liu et al., 2022; Li et al., 2024). Both the established TaqMan and SYBR Green I-based qPCR assays achieved higher detection rates than conventional PCR. Although the detection results could be determined without gel electrophoresis and imaging, the two real-time PCR assays needed precision qPCR instruments.

## 5 Conclusion

We successfully established the MIRA-LFD assay for FeChPV detection without requiring high-precision instruments. As an efficient, economical, and reliable detection method with high sensitivity and specificity, the MIRA-LFD assay is more suitable for clinical detection and provides technical support for the early prevention and control of FeChPV.

## Data Availability

The original contributions presented in the study are included in the article/**Supplementary Material**. Further inquiries can be directed to the corresponding authors.

## References

[B1] Abayli H. Can-Sahna K. (2022). First detection of feline bocaparvovirus 2 and feline chaphamaparvovirus in healthy cats in Turkey. Vet. Res. Commun. 46, 127–136. doi: 10.1007/s11259-021-09836-w 34553342 PMC8457779

[B2] Baker K. S. Leggett R. M. Bexfield N. H. Alston M. Daly G. Todd S.. (2013). Metagenomic study of the viruses of African straw-coloured fruit bats: detection of a chiropteran poxvirus and isolation of a novel adenovirus. Virology 441, 95–106. doi: 10.1016/j.virol.2013.03.014 23562481 PMC3667569

[B3] Chang W. S. Li C. X. Hall J. Eden J. S. Hyndman T. H. Holmes E. C.. (2020). Meta-transcriptomic discovery of a divergent circovirus and a chaphamaparvovirus in captive reptiles with proliferative respiratory syndrome. Viruses 12 (10), 1073. doi: 10.3390/v12101073 32992674 PMC7600432

[B4] Chong R. Shi M. Grueber C. E. Holmes E. C. Hogg C. J. Belov K.. (2019). Fecal viral diversity of captive and wild tasmanian devils characterized using virion-enriched metagenomics and metatranscriptomics. J. Virol. 93 (11), e00205-19. doi: 10.1128/JVI.00205-19 30867308 PMC6532096

[B5] Cotmore S. F. Agbandje-McKenna M. Chiorini J. A. Mukha D. V. Pintel D. J. Qiu J.. (2014). The family parvoviridae. Arch. Virol. 159, 1239–1247. doi: 10.1007/s00705-013-1914-1 24212889 PMC4013247

[B6] Cotmore S. F. Tattersall P. (2014). Parvoviruses: small does not mean simple. Annu. Rev. Virol. 1, 517–537. doi: 10.1146/annurev-virology-031413-085444 26958732

[B7] Cui H. Xu S. Xu X. Ji J. Kan Y. Yao L.. (2023b). Multienzyme isothermal rapid amplification and lateral flow dipstick combination assay for visible detection of chicken chaphamaparvovirus. Poult. Sci. 102, 103144. doi: 10.1016/j.psj.2023.103144 37839164 PMC10589884

[B8] Cui H. Zhang Z. Xu X. Zuo K. Ji J. Guo G.. (2023a). Molecular identification of carnivore chaphamaparvovirus 2 (feline chaphamaparvovirus) in cats with diarrhea from China. Front. Vet. Sci. 10. doi: 10.3389/fvets.2023.1252628 PMC10580804 37854096

[B9] Di Martino B. Di Profio F. Melegari I. Marsilio F. (2019). Feline virome-A review of novel enteric viruses detected in cats. Viruses 11 (11), 908. doi: 10.3390/v11100908 31575055 PMC6832874

[B10] Di Martino B. Lanave G. Di Profio F. Melegari I. Marsilio F. Camero M.. (2020). Identification of feline calicivirus in cats with enteritis. Transbound Emerg. Dis. 67, 2579–2588. doi: 10.1111/tbed.13605 32359195

[B11] Di Profio F. Sarchese V. Palombieri A. Fruci P. Massirio I. Martella V.. (2022). Feline chaphamaparvovirus in cats with enteritis and upper respiratory tract disease. Transbound Emerg. Dis. 69, 660–668. doi: 10.1111/tbed.14032 33559350

[B12] Du J. Wang W. Chan J. F. Wang G. Huang Y. Yi Y.. (2019). Identification of a novel ichthyic parvovirus in marine species in hainan island, China. Front. Microbiol. 10. doi: 10.3389/fmicb.2019.02815 PMC6907010 31866980

[B13] Ge Z. Carrasco S. E. Feng Y. Bakthavatchalu V. Annamalai D. Kramer R.. (2020). Identification of a new strain of mouse kidney parvovirus associated with inclusion body nephropathy in immunocompromised laboratory mice. Emerg. Microbes Infect. 9, 1814–1823. doi: 10.1080/22221751.2020.1798288 32686622 PMC7473309

[B14] Hao X. Li Y. Chen B. Wang H. Wang X. Xiao X.. (2022). Detection of FeChPV in a cat shelter outbreak of upper respiratory tract disease in China. Front. Microbiol. 13. doi: 10.3389/fmicb.2022.1064747 PMC9773189 36569076

[B15] Hargitai R. Boros Á. Pankovics P. Mátics R. Altan E. Delwart E.. (2021). Detection and genetic characterization of a novel parvovirus (family Parvoviridae) in barn owls (Tyto alba) in Hungary. Arch. Virol. 166, 231–236. doi: 10.1007/s00705-020-04862-6 33136208

[B16] Heng P. Liu J. Song Z. Wu C. Yu X. He Y. (2022). Rapid detection of Staphylococcus aureus using a novel multienzyme isothermal rapid amplification technique. Front. Microbiol. 13. doi: 10.3389/fmicb.2022.1027785 PMC9606696 36312945

[B17] Ji C. Feng Y. Sun R. Gu Q. Zhang Y. Ma J.. (2022). Development of a multienzyme isothermal rapid amplification and lateral flow dipstick combination assay for bovine coronavirus detection. Front. Vet. Sci. 9. doi: 10.3389/fvets.2022.1059934 PMC9845563 36686176

[B18] Li S. Huo X. Mu Y. Liu X. Wu J. Chen Y.. (2024). TaqMan-based real-time polymerase chain reaction for the detection of feline chaphamaparvovirus. 3. Biotech. 14, 61. doi: 10.1007/s13205-024-03917-8 PMC10850043 38344284

[B19] Li X. Wu H. Wang L. Spibey N. Liu C. Ding H.. (2018). Genetic characterization of parvoviruses in domestic cats in Henan province, China. Transbound Emerg. Dis. 65, 1429–1435. doi: 10.1111/tbed.13014 30188020

[B20] Li Y. Gordon E. Idle A. Altan E. Seguin M. A. Estrada M.. (2020). Virome of a feline outbreak of diarrhea and vomiting includes bocaviruses and a novel chapparvovirus. Viruses 12 (5), 506. doi: 10.3390/v12050506 32375386 PMC7291048

[B21] Lima D. A. Cibulski S. P. Tochetto C. Varela A. Finkler F. Teixeira T. F.. (2019). The intestinal virome of malabsorption syndrome-affected and unaffected broilers through shotgun metagenomics. Virus Res. 261, 9–20. doi: 10.1016/j.virusres.2018.12.005 30543873

[B22] Liu X. Li S. Liu X. Wang R. Xie X. Wu H.. (2022). Establishment of SYBR green I-based quantitative real-time polymerase chain reaction for the rapid detection of a novel Chaphamaparvovirus in cats. 3. Biotech. 12, 91. doi: 10.1007/s13205-022-03150-1 PMC8918419 35308811

[B23] Liu X. Wang H. Liu X. Li Y. Chen J. Zhang J.. (2020). Genomic and transcriptional analyses of novel parvoviruses identified from dead peafowl. Virology 539, 80–91. doi: 10.1016/j.virol.2019.10.013 31706163

[B24] Mohamed N. Nilsson E. Johansson P. Klingström J. Evander M. Ahlm C.. (2013). Development and evaluation of a broad reacting SYBR-green based quantitative real-time PCR for the detection of different hantaviruses. J. Clin. Virol. 56, 280–285. doi: 10.1016/j.jcv.2012.12.001 23290388

[B25] Park S. B. Chang S. (2022). Development of recombinase polymerase amplification combined with lateral flow dipstick assay to detect hemolysin gene of vibrio vulnificus in oysters. J. Food Prot. 85, 1716–1725. doi: 10.4315/JFP-21-455 35435978

[B26] Pénzes J. J. Söderlund-Venermo M. Canuti M. Eis-Hübinger A. M. Hughes J. Cotmore S. F.. (2020). Reorganizing the family Parvoviridae: a revised taxonomy independent of the canonical approach based on host association. Arch. Virol. 165, 2133–2146. doi: 10.1007/s00705-020-04632-4 32533329

[B27] Sun M. L. Lai H. Y. Chong N. Y. Liu D. F. Zhang Z. Y. Pang B.. (2021). Simple and feasible detection of hepatitis B virus via combination of multienzyme isothermal rapid amplification and lateral flow dipstick strip. Front. Mol. Biosci. 8. doi: 10.3389/fmolb.2021.763079 PMC8674754 34926579

[B28] Sun M. L. Zhong Y. Li X. N. Yao J. Pan Y. Q. (2023). Simple and feasible detection of hepatitis a virus using reverse transcription multienzyme isothermal rapid amplification and lateral flow dipsticks without standard PCR laboratory. Artif. Cells Nanomed. Biotechnol. 51, 233–240. doi: 10.1080/21691401.2023.2203198 37102677

[B29] Vibin J. Chamings A. Klaassen M. Bhatta T. R. Alexandersen S. (2020). Metagenomic characterisation of avian parvoviruses and picornaviruses from Australian wild ducks. Sci. Rep. 10, 12800. doi: 10.1038/s41598-020-69557-z 32733035 PMC7393117

[B30] Wang Y. Li W. Guo X. Zhang D. Sun J. Fu Z.. (2021). Development of SYBR Green I-based polymerase chain reaction for feline bocavirus 1 detection. 3. Biotech. 11, 61. doi: 10.1007/s13205-020-02577-8 PMC7799429 33457175

[B31] Wang Y. Yang S. Liu D. Zhou C. Li W. Lin Y.. (2019). The fecal virome of red-crowned cranes. Arch. Virol. 164, 3–16. doi: 10.1007/s00705-018-4037-x 30225519 PMC7086969

[B32] Yang S. Liu Z. Wang Y. Li W. Fu X. Lin Y.. (2016). A novel rodent Chapparvovirus in feces of wild rats. Virol. J. 13, 133. doi: 10.1186/s12985-016-0589-0 27473724 PMC4966819

