# Supplementary material for: Visible and rapid detection of feline chaphamaparvovirus using multienzyme isothermal rapid amplification and lateral flow dipstick assay
Source: Front Cell Infect Microbiol. 2025 Jan 22;15:1490948. doi: 10.3389/fcimb.2025.1490948 (PMC11794484; doi:10.3389/fcimb.2025.1490948)
Supplement: Supplementary file 1 [file Table1.docx]

Supplementary Table 1

Primers and probe used in this study.

| **Primer** | **Sequence (5′ to 3′)** | **Position^a^** | **Products** |
| --- | --- | --- | --- |
| FeChPV-1F | ATTAACAGCAAATATGATTCTAAGCAGG | 2475-2502 | 112 bp |
| FeChPV-1R | Biotin-AGACCACCTTCCTATCCAATCGTTGTTT | 2559-2586 |  |
| FeChPV-2F | ATTAACAGCAAATATGATTCTAAGCAGG | 2475-2502 | 128 bp |
| FeChPV-2R | Biotin-GTAGGAACATTTAGGGAGACCACCTTCC | 2575-2602 |  |
| Probe1-FeChPV | FAM-ATGGTGGTGTTGGGGAAAAGGAGCAAA/idsp  /CCTCTGAAACTTCAGTTC3-spacer | 2505-2549 |  |
| FeChPV-3F | GCTTACTGGCAAAACATGCCATACATATATCCT | 2683-2715 | 113bp |
| FeChPV-3R | Biotin-ATTGAAGGTATTATATGCCATCCAGTGTTTATT | 2763-2795 |  |
| FeChPV-4F | GCTTACTGGCAAAACATGCCATACATATATCCT | 2683-2715 | 142 bp |
| FeChPV-4R | Biotin-GTCTTGGAGTTATGAAATGACGCCACAAGATTG | 2792-2824 |  |
| Probe2-FeChPV | FAM-AATAATACGYATAATGTACAAGTAAGCAG/idsp  /GAAACTGGTGGAAAT-C3-spacer | 2716-2760 | / |

^a^ Genome position is based on the sequence of FeChPV prototype strain, accession No.: MN396757.

Supplementary Table 2.

Detection results of FeChPV via nested-PCR and MIRA-LFD

| Samples | positive rate | | | Statistical analysis results |
| --- | --- | --- | --- | --- |
|  | nt-PCR | MIRA-LFD | Kappa | *p* |
| Diarrhea cat | 27/417 | 29/417 | 0.962 | < 0.001 |
| Healthy cat | 3/115 | 3/115 | 1 | < 0.001 |
| Diarrheal dog | 6/342 | 6/342 | 1 | < 0.001 |
| Healthy dog | 0/132 | 0/132 | 1 | < 0.001 |
